# Dual role of autophagy in HIV-1 replication and pathogenesis

**DOI:** 10.1186/1742-6405-9-16

**Published:** 2012-05-20

**Authors:** M Scott Killian

**Affiliations:** 1 Department of Medicine, University of California San Francisco, Box 1270, 513, Parnassus Avenue, San Francisco, CA, 94143-1270, USA

**Keywords:** HIV, Autophagy, Innate immunity, T cells, Antiretroviral therapy, Pathogenesis

## Abstract

Autophagy, the major mechanism for degrading long-lived intracellular proteins and organelles, is essential for eukaryotic cell homeostasis. Autophagy also defends the cell against invasion by microorganisms and has important roles in innate and adaptive immunity. Increasingly evident is that HIV-1 replication is dependent on select components of autophagy. Fittingly, HIV-1 proteins are able to modulate autophagy to maximize virus production. At the same time, HIV-1 proteins appear to disrupt autophagy in uninfected cells, thereby contributing to CD4+ cell death and HIV-1 pathogenesis. These observations allow for new approaches for the treatment and possibly the prevention of HIV-1 infection. This review focuses on the relationship between autophagy and HIV-1 infection. Discussed is how autophagy plays dual roles in HIV-1 replication and HIV-1 disease progression.

## **Introduction**

Human immunodeficiency virus 1 (HIV-1) establishes a chronic infection that is characterized by persistent virus replication, a systemic decline in CD4+ T cell numbers, accumulating immunologic defects, and the eventual rise of AIDS-defining opportunistic infections and cancers [1]. It is increasingly evident ^1^ that autophagy, a proteolytic mechanism, plays roles in both HIV-1 replication and disease progression. This review discusses substantial findings from basic research and translational studies of autophagy and HIV-1. Emphasized is the relevance of (macro)autophagy to HIV-1 replication, anti-HIV-1 immune responses, and HIV-1 pathogenesis.

### **Overview of autophagy**

Autophagy, first described many decades ago, has been popularized by recent advances in its cellular and molecular characterization [2]. Although other systems (e.g. microautophagy and chaperone-mediated autophagy) also transport cytoplasmic material to lysosomes, macroautophagy, hereafter referred to as autophagy, is the dominant mechanism for degrading long-lived proteins and organelles [3].

**Figure 1:**
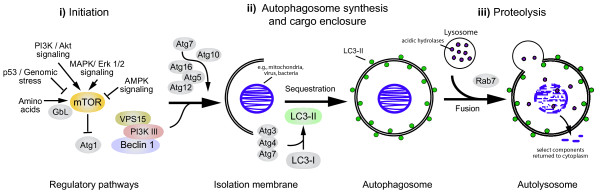
**The 3 stages of autophagy. Illustrated are distinct stages in the process of autophagy.i**) Autophagy is initiated by pathways that inactivate mTOR. **ii**) Autophagosome synthesis involves the coupling of LC3-II to the autophagosome membrane and the formation of double membrane vesicles that sequester cytoplasmic material. **iii**) The final stage of autophagy, proteolysis, entails the fusion of mature autophagosomes with lysosomes and the release of breakdown products into the cytoplasm.

Autophagy can be conceptualized as a three-stage process (Figure 1). Stage 1, the initiation of autophagy, is triggered by events that include nutrient starvation, cytokine signaling, and genomic stress. Many of these signals intersect with the mammalian target of rapamycin (mTOR) and act to reverse its inhibitory effects on autophagy [4]. Stage 2, autophagosome synthesis, involves the functions of more than 20 autophagy-related (ATG) genes (Table 1), two ubiquitin-like systems (Atg12-Atg5 and LC3-PE), and one lipid kinase signaling complex (PI3K/Beclin-1) [5]. Its end result is the formation of a double-membrane vesicle that contains cytosolic content. Stage 3, the proteolytic stage of autophagy, entails the fusion of mature autophagosomes with lysosomes [6]. Its coordination has recently been attributed to the function of the transcription factor EB (TFEB) [7]. The cytosolic contents are degraded by lysosomal acid hydrolases and then returned to the cytoplasm via channels in the autophagolysosomal membrane. Reactivation of mTOR terminates autophagy [8].

**Table 1:** **Major human autophagy**-**related genes and their functions**

**Stage of autophagy**	**Gene**	**Alternate name**	**Chromosome**	**Function in autophagy**	**References**
***Initiation of autophagy***					
	mTOR		1p36.2	Negative regulator of autophagy.	[4]
	RPTOR		17q25.3	Acts to regulate mTOR.	[9]
	ULK1	ATG1	12q24.3	Components of ULK1 protein kinase complex.	[10]
	ATG13		11p11.2		[11]
	RB1CC1	FIP200	8q11.23		[12]
	C12orf44	ATG101	2q13.13		[11]
***Autophagosome formation***					
	ATG9A,B		2q35, 7q36	Components of ATG9-WIPI complex and Vps34-beclin1 class III PI3-kinase complex.	[3,13]
	WIPI	ATG18	17q24.2		[14]
	PIK3C3	VPS34	18q12.3		[3]
	PIK3R4	VPS15	3q22.1		
	BECN1	ATG6	17q21		[15]
	ATG14		14q22.3		[15]
	UVRAG	VPS38	11q13.5		[3,16]
	Rubicon	KIAA0226	3q29		
	AMBRA1		11p11		
	ATG2A, B		11q13, 14q32		[17,18]
	ATG12		5q22	Autophagosome formation; Atg12 conjugation. Atg7 and Atg10 are E1- and E2-like enzymes respectively.	[19]
	ATG5		6q21		[18]
	ATG16L	ATG16	2q37.1		[20]
	ATG7		3p25.3		[21]
	ATG10		5q14.1		[19]
	MAP1LC3B	ATG8	16q24.2	Autophagosome maturation; LC3/Atg8 conjugation.	[3]
	GABARAP	ATG8	17p13.1		[22]
	GABARAPL2	GATE16	16q22.1		
	ATG7		3p25.3		[21]
	ATG3		3q13.2		[23]
	ATG4		Xq22.3		[24]
***Autophagosome-lysosome fusion and degradation***					
	TFEB		6p21	Transcription factor that regulates Atg and lysosomal genes.	[7]
	RAB7		3q21	Mediates fusion between autophagosome and lysosome.	[25,26]

### **Measuring autophagy**

**Figure 2:**
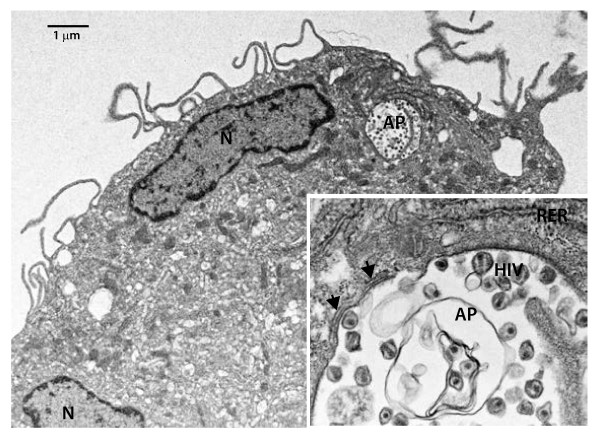
**Example of an autophagosome in an HIV-infected cell.** Shown is a transmission electron micrograph of an HIV-1_SF33_-infected monocyte-derived macrophage (L Ackerman and MS Killian, unpublished data). At higher magnification, the HIV-1 particles (roughly 0.1 μm in diameter) are clearly visible within the autophagosomal structure (inset). Abbreviations: nucleus, N; autophagosome, AP; rough endoplasmic reticulum, RER. Arrows point to the multiple membranes surrounding the autophagosome.

Several methods are commonly used to measure autophagy (Table 2) [27]. One approach is to measure, by Western blot procedures, the intracellular levels of two variants of the MAP kinase light chain 3 (LC3) protein: LC3-I and LC3-II. LC3-I is lipidated to form LC3-II and then associates with the autophagosomal membrane upon the induction of autophagy. Thus, measurement of the ratio of LC3-II to LC3-I by Western blot methods is widely used to enumerate autophagic flux; the LC3-II/LC3-I ratio increases upon the induction of autophagy and autophagosome formation. Notably, there are three mammalian isoforms of LC3: LC3A, LC3B, and LC3C. LC3B-II is the only protein known to specifically localize to the autophagosome [28]. Another tactic is to visualize and enumerate autophagosomes by electron microscopy (Figure 2). The extraordinary magnification of intracellular constituents enabled by electron microscopy allows for the direct visualization of the double membrane autophagosome structures [29]. The use of LC3-GFP fusion proteins has been helpful for measuring autophagy, allowing for streamlined fluorescent microscopy procedures [30]. Finally, kinetic RT-PCR is regularly used to measure the relative levels of autophagy-related gene transcripts such as BECN1, the gene that encodes Beclin 1 (see Table 1).

**Table 2:** General methods for measuring autophagy*

**Targeted component of autophagy**	Procedure **
Direct enumeration and quantitation of autophagosomes.Visible as double-membrane vesicles.	Electron microscopy***
LC3-II to LC3-I ratio. Provides a measurement of autophagic flux with the LC3B-II/LC3B-I ratio concomitantly increasing with autophagosome numbers.	WB****
LC3 localization. Punctate spots visible by microscopy. Total intracellular levels may increase along with autophagosome numbers.	ICC, FC, transfection of LC3 reporter plasmid followed by fluorescent microscopy or FC
Quantitation of autophagy-associated gene expression levels, e.g., BECN1.	qPCR
Quantitation of autophagy-associated protein levels, e.g., Beclin-1.	WB, ELISA
Silencing of autophagy-associated genes.	RNAi
Manipulation of autophagic flux.	Use of rapamycin, bafilomycin A1, and 3-MA

* Because the process is highly conserved among eukaryotes, these methods are broadly applicable to studies of autophagy in humans and animal models [27,31].

** Western blot (WB), flow cytometry (FC), immunocytochemistry (ICC), quantitative PCR (qPCR), RNA interference (RNAi).

*** “Gold standard” method for quantifying the number of autophagosomes.

**** “Gold standard” method for quantitating autophagic flux. Some commercially available anti-human LC3 antibodies are cross-species reactive, allowing for studies with non-human primate cells (e.g. Anti-MAP1LC3B2, cat.no. AB2970, Millipore, Billerica, MA).

Chemical compounds can also be useful for investigating the different stages of autophagy (see Figure 1). Rapamycin inhibits mTOR to induce autophagy [32]. 3-methyladenine (3-MA) inhibits class III phosphatidylinositol 3-kinase (PI3K) and thereby blocks autophagosome formation [33]. Bafilomycin A1 is a specific inhibitor of vacuolar H + ATPase that blocks the fusion between autophagosomes and lysosomes [34]. Thus, 3-MA and Bafilomycin A1 can be used to study the effects of inhibiting the early and late stages of autophagy respectively.

Studies of autophagy must be carefully evaluated with respect to the details of the assays employed and the interpretation of their results [27,28]. For example, punctate dots in fluorescent microscope images do not necessarily represent autophagosomes, as LC3 can form autophagy-independent aggregates within the cell [35]. In addition, the chemical compounds used to modify autophagic conditions, such as rapamycin, can be toxic to cells at relatively low concentrations [36]. Noteworthy is that prolonged 3-MA treatment in nutrient-rich medium is reported to promote autophagy flux in some cell lines [37]. Therefore, approaches to inhibit autophagy can actually have the opposite effect under certain conditions.

### **Autophagy and HIV-1 replication**

**Figure 3:**
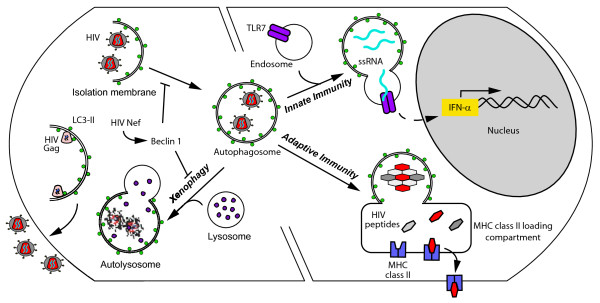
**Autophagy in HIV-1 infection.** Left) HIV-1 replication requires early autophagic events for its replication [39], perhaps because the autophagosomal membrane provides a scaffold for virion assembly. Incomplete, or fully formed virions that enter the cell via endocytosis [40], could be degraded by the autophagy (xenophagy) pathway. However, HIV-1 can inhibit the late stage of autophagy to avoid the digestion of virions within autolysosomes. Right) Autophagy is a crucial component of innate and adaptive immune responses to HIV-1 infection. Autophagy is required for the TLR7-mediated signaling of interferon-alpha (IFN-α) production by plasmacytoid dendritic cells (innate immunity) in response to HIV [41]. Autophagy also contributes to proteolytic processing for the presentation of HIV-1 peptides in the context of MHC class II (adaptive immunity).

As an obligate intracellular parasite, HIV-1 is dependent on its ability to evade intrinsic cellular defenses including xenophagy - the engulfment and destruction of intracellular microbes by autophagy (Figure 3) [38]. Discussed below are studies demonstrating that HIV-1 is able increase virus production by inducing autophagy and evading its proteolytic components.

### **Genetic studies**

At least 35 genes (see Table 1) are involved in autophagy [3,38]. Several of these autophagy-associated genes have been linked with HIV-1 replication. Using small interfering RNA (siRNA) to knock down host genes in a HeLa-derived (epithelial) cell line, members of protein-conjugation pathways involved in autophagy (ATG7, ATG8, ATG12, and ATG16L2) and lysosomal-associated genes (CLN3, LapTM5) were found to be essential for HIV-1 replication [42]. Similarly, a recent study found that the knockdown of PIK3R4, ATG4A, ATG5, or ATG16 with short hairpin RNA (shRNA) led to the inhibition of HIV-1_LAI_ replication in SupT1 cells (T cell line) without having gross effects on the cell viability [43]. However, in separate studies, treatment of HIV-infected HeLa and H9 cells (a T cell line) with rapamycin to induce autophagy did not increase HIV-1 replication [39]. Thus, additional studies are needed to better determine the relationships between the select autophagy genes and HIV replication.

### **Studies of T cells**

CD4+ T cells are the major targets of HIV-1 infection [1]. Zhou and Spector were the first to report that HIV-1 can down regulate autophagy in productively infected CD4+ T cells [44]. They found that Beclin 1 levels were substantially decreased in primary CD4+ T cells that were infected with HIV-1_MN_, an X4 strain (i.e., using the CXCR4 co-receptor), in comparison to uninfected cells and to those treated with aldrithiol-2 (AT-2) inactivated HIV-1 [44]. In addition, LC3-II levels were reduced in the HIV-infected cells as measured by confocal microscopy and Western blot procedures [44]. The inhibition of autophagy in the HIV-infected cells was found to be reversible by nutrient starvation and rapamycin [44]. This finding suggests that the inhibitory effect of HIV-1 infection on autophagy in CD4+ T cells occurs upstream of mTOR and primarily acts to block the initiation stage of autophagy (see Figure 1). Others have observed that HIV-1 infection inhibits autophagy in the MOLT-4 CD4+ T cell line [45]. Infection of MOLT-4 cells with HIV-1_NL4-3_ (X4) or HIV-1_NL4-Ad8_, an R5 variant (i.e., using the CCR5 co-receptor), caused reductions in the number of autophagosomes visible by transmission electron microscopy (TEM) and in the levels of LC3-II as measured in Western blots [45]. Notably, the LC3-I level was also reduced in the HIV-infected MOLT-4 cells, possibly reflecting the presence of a broad effect on transcription.

In contrast to the observations above, HIV-1 and HIV-2 infections have been recently reported to induce autophagy in Jurkat cells (a human T cell leukemia cell line) and primary CD4+ T cells [46]. HIV-1_MN_- and HIV-2_Rod_-infected Jurkat cells exhibited increased numbers of autophagosomes in electron micrographs and the increased expression of various autophagy-associated genes including ULK1, Atg4D, and BECN1. The inhibition of autophagy with 3-MA (concentration not stated) or through the siRNA-mediated knockdown of BECN1 resulted in decreased levels of HIV-1 RNA in the supernatants of HIV-1_MN_-infected Jurkat cell cultures. Because 3-MA can be toxic to cells (e.g., when > [0.5 mM]) and the knockdown of BECN1 can slow cell growth [43], potentially reducing HIV replication in an autophagy-independent manner, it is important to note that the cell viability and proliferation was not adequately assessed in these studies. Also reported, primary CD4+ T cells infected with HIV-1 for 3 days were found to exhibit increased Beclin-1 levels in Western blots and increased LC3 immunofluorescence [46]. In light of the findings of decreased autophagy in HIV-infected primary CD4+ T cells by Zhou and Spector [44], the observations of increased autophagy in HIV-infected Jurkat cells [46] could be explained by the presence of inherent differences in autophagy between primary T cells and some immortalized cell-lines. The observations of increased Beclin-1 and LC3 levels in the later studies of HIV-1-infected primary CD4+ T cells [46] are most consistent with the effect of the exposure to HIV (see below) rather than the consequence of HIV infection, particularly as the percent of HIV-infected cells ^2^ was not established in those studies.

Importantly, HIV-1 also modifies autophagy in uninfected bystander CD4+ T cells. In a seminal paper, Espert et al. reported that the accumulation of autophagosomes and Beclin 1 in umbilical cord blood CD4+ T lymphocytes was similarly induced by i) HEK.293 cells transfected to express HIV-1 Env, ii) rapamycin, and iii) CEM T cells infected with HIV-1_NL4-3_[47]. Autophagosomes did not accumulate in CD4+ T cells treated with the drugs 3-MA and AMD3100 (a CXCR4 antagonist). Also described in this study was that the Env-induced autophagy preceded apoptotic cell death (see Autophagy and HIV-1 disease progression). In follow-up studies, this group determined that the fusion activity of the HIV-1 envelope glycoprotein gp41 was primarily responsible for this effect [48] and that more than 30 candidate proteins are associated [49]. These proteins were largely involved in degradation processes, redox homeostasis, metabolism and cytoskeleton dynamics, and linked to mitochondrial functions. Espert et al. have more recently reported that R5 Env also induces autophagy and cell death in uninfected CD4+ T cell lines [45]. Thus, the effect of HIV-1 Env on autophagy in CD4+ T cells does not appear to be co-receptor specific. Env triggers a broad decrease in protein synthesis that may act to induce autophagy by reducing levels of the inhibitory protein mTOR [49]. The contrasting observations of inhibited autophagy in HIV-1 infected CD4+ T cells and elevated autophagy in bystander CD4+ T cells suggest that productive infection can reverse the enhancing effect of HIV-1 Env on autophagy in uninfected CD4+ T cells.

### **Studies of macrophages**

Macrophages are another important cellular reservoir of HIV-1 [50]. Two groups have reported similar findings regarding autophagy in HIV-infected monocyte-derived macrophages (MDM) [39,45]. In agreement with studies of HeLa cells [42], the induction of autophagy appears to be necessary for HIV-1 replication in MDM. The infection of MDM, upon exposure to HIV-infected cells, is associated with increased numbers of autophagosomes [45]. Interestingly, HIV was detected in the cells with moderate, but not high, numbers of autophagosomes [45]. HIV-infected MDM, cultured in the presence of 3-MA, exhibited substantial reductions in the production of both R5 and X4 viruses [45]. The siRNA-mediated knockdown of Beclin 1 and Atg7 also diminished virus production in HIV-1_SF162_-infected MDM and U937 cells (a myelomonocytic cell line) [39]. Conversely, treatment of MDM, THP-1 (a myelomonocytic cell line), and U937 cells with rapamycin to induce autophagy increased HIV-1 production [39]. The late stages of autophagy (i.e., lysosome fusion and proteolysis) reduce HIV-1 production, as evidenced by the effects of bafilomycin A1 [39]. While most studies of macrophages indicate that HIV-1 promotes autophagosome formation and inhibits the late proteolytic stage of autophagy, decreased LC3-II levels in HIV-1-infected U937 cells have been reported [44]. Thus, the effects of HIV-1 on autophagy in monocytic cell lines can differ from those found in MDM.

Mechanisms enabling HIV-1 to subvert autophagy in macrophages have been elucidated [39]. Demonstrated is that HIV-1 Gag and Nef interact with the autophagy proteins LC3 and Beclin 1 respectively. The colocalization of Gag with LC3 suggests that autophagy plays a role in the biosynthesis, processing, or assembly of HIV-1 intermediates [39]. Alternatively, the Gag/LC3 colocalization could reflect the targeting of Gag for autophagic degradation. The association between Gag and LC3 appears to be unique to monocytic cells [39]. In binding protein complexes containing Beclin 1, Nef is able to inhibit the proteolytic stages of autophagy and thereby prevent the destruction of HIV-1 intermediates [39]. Thus, HIV-1 Nef acts as an “antiautophagic maturation factor” [39]. The Nef ^174^DD^175^ motif that is needed for CD4 downmodulation [51] is required for its interaction with Beclin 1 [39]. Recently, Nef has also been shown to interact with immunity-associated GTPase family M (IRGM) to induce autophagy in macrophages [52]. These findings indicate that Nef can have the dual function of initiating autophagy and inhibiting its maturation. Unexplained is the normal replication and cytopathicity exhibited by some Nef-deleted HIV-1 isolates *in vitro* and *in vivo*[53,54].

Another distinction between CD4+ lymphocytic and monocytic cells is the effect of HIV-1 Env on autophagy in bystander cells. In contrast to its effect on CD4+ T cells, HIV-1 R5 and X4 Env, when expressed by transfected HEK.293 cells, do not trigger uninfected human monocytic leukemia THP1 cells, MDM, or U937 to undergo autophagy [45]. This distinction could explain the occurrence of CD4+ T cell losses amidst relatively stable monocyte levels in HIV-infected individuals [55].

HIV-1 Tat can have an indirect effect on autophagy in macrophages. In healthy macrophages, autophagy is induced by the pro-inflammatory cytokine interferon-gamma (IFN-γ) [56]. However, pretreatment of monocyte-derived macrophages with HIV-1 Tat, inhibits the stimulatory effect of IFN-γ on autophagy and impairs the antimicrobial functions of the cells [56]. The underlying mechanism involves the ability of Tat to block STAT1 phosphorylation [56]. In other studies, Tat has been found to inhibit autophagy in uninfected macrophages by increasing Akt, Src, and IL-10 production, leading to the silencing of STAT3 and inhibition of autophagy [57].

To summarize, the studies above indicate that HIV-1 proteins disrupt autophagy in HIV-infected and uninfected cells (Table 3). The effects of HIV-1 on autophagy are cell-type specific and could be associated with the observed differences in infectivity, virus replication kinetics, and cytopathicity among CD4+ cells of different hematopoietic lineages. In this regard, studies of cell-lines can be misleading with respect to the relationships between HIV and autophagy in primary cells.

**Table 3:** Relationships between HIV-1 proteins and autophagy

**HIV-1 protein**	**Relationship with autophagy**	**References**
Gag	In macrophages: Gag colocalizes with LC3, perhaps to promote virion assembly.	[39,45]
Env	In bystander T cells and neuronal cells: Env induces autophagy and promotes autophagic T cell death.	[47,58]
Nef	Nef interacts with IRGM to induce autophagy. Nef also acts as an "antiautophagic maturation factor" and blocks the late proteolytic stage of autophagy.	[52,39]
Tat	In macrophages: Tat blocks IFN-γ-induced LC3 expression and inhibits autophagy.	[59]
	In bystander HUVEC*: Tat increases autophagy.	[60]

* Human umbilical vein endothelial cells.

### **Autophagy and anti-HIV-1 immune responses**

Distinguishing features of progressive HIV-1 infection include impaired innate and adaptive immune responses and hyper-immune activation [1,61]. Autophagy is essential for the functionality of innate and adaptive immune responses (see Figure 3) and the maintenance of self-tolerance [38,62]. Thus, autophagy can play important roles in immune cell functions that have direct relevance to HIV-1 infection.

### **Innate immunity**

Innate immune responses provide the earliest host defense against microbial invasion [63]. Cells of the innate immune system use pattern recognition receptors (e.g. Toll-like receptors [TLRs] and nucleotide-binding oligomerization domains [NODs]) to identify highly conserved pathogen-associated molecular patterns (PAMPs, e.g., unmethylated CpG motifs and viral single-stranded RNA) [64]. The cell types of the innate immune system that can exhibit direct anti-HIV-1 activity include plasmacytoid dendritic cells (pDCs), natural killer (NK) cells, and monocytes/macrophages. While pDCs are scarcely present in the blood (< 10 cells per μl), they are the major type-1 interferon (IFN-α) producing cells [65]. pDCs secrete large amounts of IFN-α in response to HIV-infected cells and thereby suppress HIV-1 replication in those cells [66]. The recognition of HIV-infected cells by pDCs appears to be primarily mediated by TLR7, a receptor for single-stranded RNA [67]. Importantly, the production of IFN-α by pDCs in response to TLR7 signaling is dependent on autophagy [68,69]. Furthermore, pDCs produce IFN-α in response to infectious or AT-2 inactivated HIV-1_MN_ through the induction of autophagy following TLR7 signaling [41]. NK cells are activated by pDCs responding to HIV-1 [70] and the ability of NK cells to lyse HIV-infected target cells is enhanced by IFN-α [71]. The activation of macrophages via innate biosensors to secrete anti-HIV cytokines such as the β-chemokines and the macrophage-derived anti-HIV factor (MDAF) [72] can require autophagy [73]. Consequently, autophagy is crucial for suppression of HIV-1 replication by the soluble factors and cytolytic functions of the innate immune system.

### **Adaptive immunity**

In addition to their roles in innate immunity, dendritic cells (DCs) and macrophages promote adaptive immune responses by surveying proteins and secreting cytokines in response to pathogens [74]. In this regard, autophagy can contribute to the processing and presentation of viral peptides in the context of major histocompatibility complex (MHC) class I and II molecules. Autophagy has been observed to enhance the presentation of HSV-1 antigens on the MHC-I molecules of macrophages [75]. In contrast, the inhibition of autophagy in DCs by 3-MA prevents the presentation of HIV-1 antigens on MHC-II, but not cross-presentation on MHC-I to CD8+ T cells [76]. Also, treatment of DCs with 3-MA to inhibit autophagy results in reduced MHC-II expression and impairs antigen presentation of respiratory syncytial virus (RSV) [77]. In other studies, most Influenza A virus antigens were presented to CD4+ T cells by MHC-II on DCs without a requirement for autophagy [78]. These heterogeneous observations suggest that the contribution of autophagy to antigen presentation via MHC-I and II molecules could be pathogen and cell-type specific.

The cytokines produced by DCs and macrophages function to regulate autophagy in other immune cells by intersecting with pathways upstream of mTOR (see Figure 1) [62]. In general, Th1 cytokines (e.g. IFN-γ) upregulate autophagy, while Th2 cytokines (e.g. IL-4 and IL-13) abrogate this process [79,80]. Notably, an intense cytokine storm occurs during acute HIV-1 infection and large amounts of IFN-γ, TNF-α, and IL-10 are produced by T cells throughout the course of disease progression [81]. Thus, the cytokine response to HIV-1 infection can influence autophagy in distal cells and tissues and thereby have pathogenic consequences (see below).

### **Self-tolerance**

The tight regulation of antiviral immune responses is necessary to prevent autoimmunity. The autophagic mechanisms used by DCs to direct adaptive immune responses (see above) are also used to promote self-tolerance. This function of autophagy is exemplified in murine models. Athymic mice implanted with Atg5^−/−^ thymus tissue exhibit inflammation of the gut (colitis) and other organs, indicating that autophagy is crucial for thymic selection and self- tolerance [82]. Implicating a role for autophagy in human autoimmune disorders are findings that 1) polymorphisms in NOD2 and ATG16L1 are associated with susceptibility to the inflammatory gut disorder Crohn's disease [83], and 2) the interaction between the two proteins encoded by these genes is essential for normal autophagy in DCs [84,85]. The dysregulation of autophagy by viral proteins, perhaps due to Env-mediated effects on bystander CD4+ T regulatory cells, could contribute to aspects of autoimmunity observed in HIV-1 infection [86].

### **Autophagy and HIV-1 disease progression**

As a general homeostasis mechanism, autophagy functions to maintain the health of cells and tissues. Aberrant autophagy has been implicated in a variety of neurodegenerative disorders, cancers, and autoimmune diseases [87]. Hence, the dysregulation of autophagy by HIV, as discussed below, could play a role in the broad pathology of HIV-1 infection (Table 4).

**Table 4:** Roles of autophagy in HIV-1 infection, pathogenesis, and treatment

**Topic**	**Observations**	**References**
HIV-1replication	In HeLa cells, autophagy-associated genes are necessary for HIV-1 replication.	[42]
	In CD4+ T cells, HIV-1 inhibits autophagy as evidenced by decreased autophagosome numbers and reduced levels of Beclin 1 and LC3 II.	[44]
HIV-1pathogenesis	In macrophages, early nondegradative stages of autophagy promote HIV-1 replication. HIV-1 Gag interacts with LC3 to elevate these stages. The late proteolytic stages of autophagy inhibit HIV-1 replication. Nef interacts with Beclin 1 to inhibit these stages	[44,45]
	In bystander T cells, HIV-1 Env induces autophagy and the accumulation of Beclin1 in uninfected CD4+ T cells. This event leads to apoptosis.	[46,47]
	Bystander macrophages do not undergo Env-mediated autophagy. HIV-1 inhibits autophagy in bystander macrophage/monocytic cells through an Akt-dependent pathway.	[57]
	In dendritic cells, HIV-1 capture down-regulates autophagy and immunoamphisomes in monocyte-derived dendritic cells, impairing innate and adaptive immune responses. Plasmacytoid dendritic cells produce IFN-α in response to infectious or noninfectious HIV-1 through autophagy-dependent TLR7 signaling. This response could promote chronic immune activation.	[41,76]
	Neurotoxicity. The dysregulation of autophagy is a feature of neuroAIDS. The brains of persons with HIV-1 encephalitis exhibit increased levels of autophagic proteins and autophagosomes.	[88,89]
Treatment *	Antiretroviral therapy. HIV-1 protease inhibitors induce autophagy in cancer cells. Clinical concentrations of EFV induce autophagy and, in particular, mitophagy in hepatic cells. ddI treatment restores neuronal LC3 expression in the brains of FIV-infected animals.	[90,91]
	Vitamin D. It has been observed that HIV-infected individuals have reduced levels of the hormonally active form of vitamin D and that this compound has autophagy-dependent anti-HIV-1 effects on macrophages.	[36]

* Abbreviations: efavirenz, EFV; feline immunodeficiency virus, FIV; didanosine, ddI.

### **Persistent virus replication and CD4+ T cell loss**

HIV-1 establishes a chronic viral infection with persistent virus replication that is the underlying cause of HIV-1 disease progression [1]. As discussed (see Autophagy and HIV-1 replication), HIV-1 subverts autophagy to promote virus replication in the infected cell. Also, the importance of autophagy in antiviral immunity was reviewed (see Autophagy and anti-HIV-1 immune responses). Because the primary targets of HIV-1 infection are CD4+ cells that play key roles in antiviral immunity, the dysregulation of autophagy in these cells further promotes the chronicity of HIV-1 infection. As described above, HIV-1 Env promotes hyper-autophagy in bystander CD4+ T lymphocytes. This effect is associated with increased apoptotic cell death and is proposed to be a major mechanism for CD4+ T cell loss [47]. During acute HIV-1-infection, massive losses of CD4+ T cells occur in gastrointestinal tissues [92,93], perhaps due to the increased sensitivity of these cells to Env-mediated autophagic cell death. Conceivably, elite controllers of HIV-1 infection, who exhibit undetectable viral loads and maintain stable CD4+ T cell counts [94], differ in their regulation of autophagy. In this regard, the restriction of virus replication by xenophagy could be an important contributor to HIV-1 latency.

### **Opportunistic infections and cancers**

The impairment of autophagy in cells of the innate and adaptive immune systems could facilitate the rise of opportunistic infections. Irregularities in the dendritic cell compartment become prevalent with progression to AIDS [1], including a decline in pDC number and function [79,95]. Thus, disruption of autophagy in dendritic cells as some studies suggest, could be an important contributor to HIV-1 disease progression. Monocyte-derived dendritic cells exposed to HIV-1 exhibit reduced LC3-II expression, decreased TLR responses, and impaired antigen presentation functions [76]. These inhibitory effects of HIV-1 on autophagy in DCs were mediated by Env-induced mTOR signaling [76]. Also, the interaction between HIV-1 Nef and Beclin-1 (discussed above) could inhibit autophagic pathways that protect against tumorigenesis [96]. Thus, by inhibiting autophagy-dependent mechanisms in DCs, HIV-1 could allow for opportunistic infections and cancers to evade innate and adaptive immune responses.

### **Neurodegenerative disease**

The clearance of misfolded and aggregate proteins via autophagy plays a protective role against neurodegenerative disorders such as Huntington’s and Parkinson’s disease [97,98]. Defects in this housekeeping function of autophagy are linked with HIV-associated neurologic diseases. Autophagic markers are moderately increased in brain tissues of persons having HIV-encephalitis (HIVE) and HIV-associated dementia (HAD) in comparison to HIV-infected brains having no impairment [58,88]. HAD is associated with increased levels of a byproduct of CXCL12 (i.e., SDF-1), a chemokine that blocks neuronal autophagy [89,99]. Soluble factors in cultures of simian immunodeficiency virus (SIV)-infected microglia also inhibit neuronal autophagy [100]. In other studies, the exposure of neuronal cells to HIV-1 gp120 resulted in increased Beclin 1, LC3-II and ATG5 levels [58]. This bystander effect of HIV-1 Env that increases autophagy in neuronal cells is similar to the one described above for CD4+ T cells (see Autophagy and HIV-1 replication).

In addition to Env, the HIV-1 transactivator protein, Tat, can also disrupt autophagy in the neurological system. Tat is detectable in the blood and cerebrospinal fluid of HIV-infected individuals [101] and has been shown to be cytotoxic to human brain microvascular endothelial cells [102]. Human umbilical vein endothelial cells (HUVEC), when exposed to cell culture medium conditioned by HeLa-Tat cells, exhibit increased levels of Nox4-dependent H_2_O_2_ production, endoplasmic reticulum (ER) stress, and autophagy [60]. Thus, circulating Tat may contribute to HIV-1 neuropathogenesis through an autophagy-dependent mechanism.

### **Cardiovascular disease and frailty**

Receiving increasing attention is evidence that HIV-1-infected individuals age at a faster rate than others [103]. In this regard, autophagy may contribute to the increased rates of cardiovascular disease (CVD) and frailty observed in HIV-infected individuals. Hyper-autophagy has an important role in several types of cardiomyopathy by functioning as a death pathway [104]. CVD, particularly congestive heart failure, is strongly associated with a frailty phenotype (e.g., increased weakness, slowness, exhaustion, anergy, and weight loss) that becomes increasingly prevalent among older adults [105,106]. Independent of age, a strong inverse correlation exists between frailty and CD4+ T cell counts among HIV-infected individuals [107]. Thus, increased autophagic CD4+ T cell death due to Env-mediated effects [47] could potentially contribute to increased frailty. Also, autophagy influences longevity in eukaryotic organisms [108] and therefore aberrant autophagy could be an important frailty factor in the context of HIV-1 infection.

### **Implications for autophagy therapeutics**

Irregular autophagy (e.g. Env-mediated hyper-autophagy), as a contributor to HIV-1 disease progression, could be therapeutically managed using a variety of pharmacologic agents (Table 5). Autophagy modifiers are being clinically evaluated for the treatment of Huntington's disease [109], renal cell carcinoma [110], aging [111], and other autophagy-related disorders [112]. Autophagy enhancing candidates include clonidine, minoxidil, verapamil, and STF-62247 [109,110]. Also, the protease inhibitors nelfinavir and saquinavir are under evaluation for their autophagy-enhancing activities [90,113]. In this regard, autophagy needs further study in HIV-infected subjects receiving protease inhibitors. Of note, very low concentrations of rapamycin (e.g., < 1 nM) can have anti-HIV-1 activity *in vitro*[114,115]. This finding makes rapamycin an attractive candidate for evaluation in the treatment of HIV-1 infection [116]. Moreover, recent evidence suggests that the autophagy promoting effects of vitamin D could be of therapeutic benefit to HIV-infected individuals [36]. Because HIV-1 can require autophagy for virus replication and this process becomes induced upon exposure to HIV, drugs that inhibit autophagy could potentially be used to lower HIV replication and to reduce hyper-autophagy levels. Candidate drugs include wortmannin and other PI3K inhibitors that are being evaluated for their potential clinical use as autophagy blockers [117].

**Table 5:** Pharmacologic modifiers of autophagy

**Drug category**	**Drug or reagent**	**Mechanism**	**References**
***Inducers and enhancers of autophagy***			
	Rapamycin*	Inhibits mTOR signaling.	[4]
	Carbamazepine	Inhibition of inositol monophosphatase.	[118]
	Lithium	Inhibition of inositol monophosphatase.	[119]
	Digoxin	Undetermined.	[120]
	Vitamin E	Increases phosphorylation of mTOR substrates.	[121]
	Verapamil	Reduces calcium flux into the cell.	[109]
	Clonidine	Reduces cyclic adenosine monophosphate (cAMP).	[109]
	Trehalose	mTOR independent mechanism.	[122]
	Tamoxifen	Increases the intracellular level of ceramide.	[123]
	Niclosamide	Inhibits mTOR signaling.	[124]
	Rottlerin	Inhibits mTOR signaling.	[124]
	Amiodarone	Inhibits mTOR signaling.	[124]
***Inhibitors of autophagy***			
	Chloroquine	Blocks the fusion of autophagosomes with lysosomes.	[125]
	Verteporfin	Inhibitor of autophagosome accumulation.	[126]
	3-Methyladenine*	Inhibitor of class III PI3K (Vps34).	[33]
	Bafilomycin A1*	Ion Channel Inhibitor; V-ATPase inhibitor.	[34]
	Wortmannin	PI3K inhibitor.	[127]
	LY294002	PI3K inhibitor.	[127]
	Leupeptin	Inhibitor of serine and cysteine proteases.	[128]
	Asparagine	Prevents transfer of autophaged material to lysosomes.	[129]

* Commonly used in basic research studies.

In light of the contributions of autophagy to classical antigen presentation and innate pathogen sensory mechanisms, autophagy modifiers could also be helpful for an HIV-1 vaccine strategy. Indeed, the observation that rapamycin-enhanced autophagy increases antigen presentation by DCs and macrophages opens novel approaches for boosting adaptive immunity in immunocompromised individuals or in the context of vaccination [130]. Furthermore, the potential effects of exposure to HIV-1 proteins, such as gp120, on autophagy (see Table 3) should be evaluated in the context of vaccine studies.

## **Conclusions**

Many questions remain pertaining to the relationship between autophagy and HIV-1 infection. Yet unknown is whether or not the ability of HIV-1 to promote autophagy in bystander CD4+ T cells renders those cells more susceptible to HIV-1 infection. Also unexplained are the differential effects of HIV-1 on autophagy in macrophages and CD4+ T cells. In addition to the need for further basic research, translational studies are needed to establish the magnitude of the effects of HIV-1 on autophagy in HIV-1 infected individuals. Moreover, few studies have evaluated autophagy in animal models of HIV infection.

In summary, the importance of autophagy in HIV-1 infection is becoming increasingly clear. Direct effects of HIV-1 on autophagy include the subversion of autophagy in HIV-infected cells and the induction of hyper-autophagy in bystander CD4+ T cells. Because HIV-1 targets key cytokine-producing and immunoregulatory cells, its disruption of autophagy in these cells can have broad pathogenic consequences. Indeed, autophagy appears to play a dual role in HIV-1 infection and disease progression.

### **Endnotes**

^1^ An April 2012 search of http://www.pubmed.gov using the terms “HIV AND autophagy” returned 55 entries in the database (Killian MS, independent observation).

^2^ With typical procedures and HIV-1 stock concentrations (e.g. 1 μg/ml p24 equivalents), the frequency of productively HIV-infected primary CD4+ T cell blasts remains relatively low at day 3 post-infection e.g., generally less than 25% of the cells exhibit intracellular p24 or CD4 down-modulation (Killian MS, independent observation).

## Competing interest

The author declares to have no competing interests.
